# Autophagy activation by dietary piceatannol enhances the efficacy of immunogenic chemotherapy

**DOI:** 10.3389/fimmu.2022.968686

**Published:** 2022-08-01

**Authors:** Shuang Wang, Guangsuo Wang, Weiqing Wu, Zhenglei Xu, Jing Yang, Min Cao, Qi Wang, Jigang Wang, Chuanbin Yang, Wei Zhang

**Affiliations:** ^1^ Department of Geriatrics, Shenzhen People’s Hospital, The Second Clinical Medical College, Jinan University, The First Affiliated Hospital, Southern University of Science and Technology, Shenzhen, China; ^2^ Department of Thoracic Surgery, Shenzhen People’s Hospital, The Second Clinical Medical College, Jinan University, The First Affiliated Hospital, Southern University of Science and Technology, Shenzhen, China; ^3^ Department of Health Management, Shenzhen People’s Hospital, The Second Clinical Medical College, Jinan University, The First Affiliated Hospital, Southern University of Science and Technology, Shenzhen, China; ^4^ Department of Gastroenterology, Shenzhen People’s Hospital, The Second Clinical Medical College, Jinan University, The First Affiliated Hospital, Southern University of Science and Technology, Shenzhen, China; ^5^ Shenzhen People’s Hospital, The Second Clinical Medical College, Jinan University, The First Affiliated Hospital, Southern University of Science and Technology, Shenzhen, China; ^6^ Artemisinin Research Center, Institute of Chinese Materia Medica, Chinese Academy of Chinese Medical Sciences, Beijing, China; ^7^ Integrated Chinese and Western Medicine Postdoctoral Research Station, Jinan University, Guangzhou, China

**Keywords:** immunogenic cell death (ICD), autophagy, transcription factor EB (TFEB), endoplasmic reticulum (ER) stress, piceatannol

## Abstract

Immunogenic cell death (ICD) promotes the immune antitumor response *via* releasing damage-associated molecular patterns (DAMPs) from dying tumor cells. The induction of autophagy improves the efficacy of multiple immunogenic chemotherapies. Here, we show that piceatannol, a dietary phenolic compound that is widely distributed in multiple fruits and vegetables such as grapes, blueberries, and mushrooms, induces autophagy and enhances oxaliplatin (OXA)-induced anticancer immune response. Specifically, piceatannol enhanced OXA-induced release of DAMPs, several key hallmarks of ICD including ATP release, cell surface exposure of calreticulin, and high-mobility group box 1 (HMGB1) release. Mechanistically, piceatannol promoted autophagy *via* activating TFEB/TFE3, two key transcription factors of the autophagy-lysosome pathway, and inhibiting autophagy attenuated piceatannol plus OXA-induced ATP release. Furthermore, piceatannol induced endoplasmic reticulum stress, which is critical for its role in enhancing OXA-induced cell surface exposure of calreticulin, another key hallmark of ICD. Consistently, the combination of piceatannol with OXA promoted the anticancer effects in immunocompetent mice. Taken together, our results indicate the importance and great potential of dietary piceatannol in cancer immunotherapy. Therefore, piceatannol may be used as an ICD enhancer that improves the efficacy of chemotherapeutics such as OXA in cancer treatment with minimized toxicity.

## Introduction

In certain circumstances, regulated cell death stimulates an inflammatory response, including the recruitment and activation of macrophages, neutrophils, and other immune cells, which finally drives both innate and adaptive immune responses (1, 2). Immunogenic cell death (ICD) is now referred to as such a kind of regulated cell death (1, 2). A key characteristic of ICD is the release of danger-associated molecular patterns (DAMPs) by tumor cells, which include ATP, calreticulin (CALR), and high mobility group box 1 (HMGB1) (3). DAMPs bind to their relevant pattern recognition receptors (PRRs) that are expressed in dendritic cells (DCs) to accelerate the maturation of DCs, and the proper presentation of tumor antigens to T cells, thus promoting long-term antitumor immunity efficacy (4). Upon ICD induction, extracellular ATP released from the nucleus serves as a “find-me” signal that can be sensed by purinergic receptor P2Y2 on dendritic cells (DCs) and promotes the recruitment of myeloid cells to the site of ICD. HMGB1 is a highly abundant nuclear protein that can be secreted during ICD (5), though the underlying mechanism is not completely understood. HMGB1 promotes the processing and presentation of tumor antigens and exerts immunostimulatory effects *via* binding to several pattern recognition receptors (PRRs) such as RAGE (advanced glycation end-products), toll-like receptor 2 (TLR2), and toll-like receptor 4 (TLR4) that are expressed inside DCs. CALR is an endoplasmic reticulum (ER)-resident protein. CALR migrates from ER to cell surface during ICD (2). Cell surface CALR serves as a “eat me” signal and enhances phagocytes to engulf dead cells *via* interaction with its receptor CD91. The immunogenicity of CALR also depends on its role in promoting type I interferons secretion by DCs. A key step for cell surface exposure of CALR is the loss of ER homeostasis and the activation of the unfolded protein response (UPR) pathway, particularly, the eukaryotic initiation factor 2α (eIF2α)-protein kinase R (PKR)-like ER kinase (PERK) branch (6–8).

The interaction of these representative DAMPs with their relevant PRRs that are expressed on DCs favors the attraction, activation, and maturation of DCs, enhances the presentation of tumor antigens to T cells and elicits T cell immune response, which eventually induces the long-term protective antitumor immunity (2, 9) related to immunogenic cell death are differentially triggered by clinically relevant chemotherapeutics in lung adenocarcinoma cells. Therefore, inducing ICD represents a unique and promising benefit for cancer therapy.

Recently, a variety of ICD inducers have been identified, which include oncolytic viruses, radiotherapy, photodynamic therapy, and importantly several classic chemotherapeutic drugs such as anthracyclines, oxaliplatin (OXA), and mitoxantrone (1, 2). As mentioned above, during chemotherapy, the release of ATP is critical for effective ICD (10). Autophagy is a conserved pathway for the degradation of protein aggregates (11). Increasing evidence has suggested that autophagy is required for ATP release in the context of anthracyclines-induced ICD. Specifically, autophagy deficiency compromised anthracyclines-induced ATP release and subsequently therapeutic immune response (12). Moreover, a variety of ‘caloric restriction mimetics’ including hydroxycitrate and short-time starvation induce ATP release through activating autophagy and thus enhance the efficacy of immunogenic drugs (13, 14). Hence, exploration of autophagy activators that serve as promising ICD amplifiers shows potential efficacy in improving the immunological and therapeutic outcomes of chemotherapeutic drugs such as OXA.

Motivated by these discoveries, we hypothesized that proper evoking of ICD hallmarks through a suitable combinatorial antitumor immunotherapy using an ICD inducer along with an autophagy enhancer is capable of enhancing the immunogenic eradication of cancer cells. Thereby, we intended to identify natural small molecules that can enhance the ICD-induced antitumor effects of OXA. Here, we identify that piceatannol (*trans*-2,3′,4’, 5-tetrahydroxystilbene, Pic), a dietary polyphenolic compound that is widely distributed in fruits and vegetables such as red grapes, blueberries (15), and mushrooms (16, 17), is a novel autophagy activator *via* regulating transcription factor EB (TFEB) and TFE3. We further determined its efficacy in enhancing OXA-induced ICD effects and elucidate the underlying mechanisms. We also evaluated the roles of piceatannol in enhancing OXA-induced anticancer immune responses in immunocompetent mice. Overall, our findings suggest that dietary piceatannol is an ICD amplifier that may be useful for potential anti-cancer therapy.

## Materials and methods

### Chemicals and reagents

Chloroquine (C6628) was obtained from Sigma-Aldrich. Oxaliplatin (OXA, S1224), and quinacrine (S5435) were purchased from Selleckchem. Torin 1 (2273-5) was ordered from BioVision. Anti-ATF4 (CST 11815S), anti-CHOP antibody (2895), anti-eIF2α antibody (9722), anti-p-eIF2α (Ser51) (9721S), anti-Histone H3 (9715S), anti-ATG5 antibody (12994), and anti-PERK (5683) antibodies were obtained from Cell Signaling Technology (CST). An anti-LC3 antibody (NB100-2220) was obtained from Novus Biologicals. An anti-GAPDH antibody (GTX100118) was ordered from GeneTex. Anti-TFE3 antibody (HPA023881), and anti-Flag (F3165) antibody were ordered from Sigma-Aldrich. Anti-TFEB antibody (303-673A) was ordered from Bethyl Laboratories. Anti-HMGB1 (ab18256), and anti-Calreticulin (ab92516) antibodies were ordered from Abcam. Goat anti-rabbit (111-035-003), and goat anti-mouse (115-035-003) secondary antibodies were products of Jackson ImmunoResearch. Alexa Fluor 594 goat anti-rabbit IgG (A-11012), Alexa Fluor 488 goat anti-rabbit IgG (A-11034), and Alexa Fluor 488 (A-11008) goat anti-rabbit secondary antibodies were ordered from Invitrogen.

### Cell culture

U2OS cells and MCA205 cells originating from ATCC were cultured in DMEM (Gibco, C11995500BT) medium containing 10% FBS (Hyclone, SH30256.01), 100 mg/mL streptomycin, and 100 U/mL penicillin and incubated at 37°C in 5% CO_2_ humidified incubators.

### Western blotting

Western blotting was conducted according to previous studies (18–20). Briefly, intracellular total proteins were extracted with RIPA lysis buffer (CST 9806) containing protease inhibitor mixture and phosphatase inhibitor. The cytoplasm and the nuclear proteins were extracted as previously described (18, 19). SDS-PAGE (10 to 15%) was used for separation of protein samples, followed by transferring of membrane and blotting with indicated primary antibodies and relevant secondary antibodies. SuperSignal™ West Femto Maximum Sensitivity Substrate (Thermo 34094) and ChemiScope 6000 Touch (Clin X) were used to detect relevant protein bands. ImageJ software was used for protein quantification.

### ATP release assays

Intracellular ATP was stained with 5 μM quinacrine and followed by Hoechst staining of nucleus as described previously. Images were acquired by a fluorescence microscope. Intracellular ATP contents were quantified using ImageJ software.

### Cell transfection

For the overexpression experiment, cells were transfected with Lipofectamine 3000 (L3000015, Thermo). For knockdown expression, cells were transfected with lipofectamine™ RNAiMAX Transfection Reagent (13778075, Thermo) and indicated siRNA for 48 h, followed by drug treatment. The relevant siRNAs sequences for each human gene are as follows: TFEB 5’- CUACAUCAAUCCUGAAAUG-3’; TFE3, 5’-GGAAUCUGCUUGAUGUGUA-3’; PERK, 5’-GGAACGACCTGAAGCTATA-3’; ATG5 5’- CAACTTGTTTCACGCTATA-3’.

### Immunofluorescence

For HMGB1 detection, cells were fixed with 4% PFA, permeabilized with Triton X-100 (0.25%), and incubated with an anti-HMGB1 antibody followed by staining with Alexa Fluor 488 secondary antibody. Slides were visualized using a Leica TCS SP8 STED microscope. Images were quantified using ImageJ software.

For tf-LC3, CALR, TFEB, and TFE3 staining, cells were transfected with tf-LC3 (Addgene 21074), Flag-TFEB (19), GFP-TFE3 (Addgene, 38120), and Calreticulin-RFP-KDEL (21) plasmids using Lipofectamine 3000 (Thermo, L3000015) for 48 h, followed by treating cells with indicated drugs for another 24 h. After fixation, cells were stained with indicated antibodies, and nucleus was stained with DAPI. The images were captured utilizing a Leica TCS SP8 STED microscope. Images were quantified using ImageJ software.

### Flow cytometry analysis of CALR surface exposure

Cells were collected and stained with anti-calreticulin (CALR) antibody for 30 min on ice before being incubated with Alexa Fluor^®^488 secondary antibody for 30 min at room temperature. Cells were fixed with 4% PFA in PBS for flow cytometric analysis.

### Animal experiments

Male C57BL/6J mice at the age of 6-8 weeks from Gempharmatech Co., Ltd were housed in an SPF house with a regular 12 light/dark cycle provided with food and water ad libitum. Mice were inoculated with 5X10^5^ MCA205 cells according to previous reports. When tumors appeared palpable (around 7 days), mice were intraperitoneally injected with piceatannol (30 mg/kg). One and two days later, OXA (10 mg/kg) and piceatannol (30 mg/kg) were injected into mice, respectively. On the following days, piceatannol (30 mg/kg) was given three times per week. Tumor growth and mice weights were recorded every two days. Mice were sacrificed at the endpoint or when signs of obvious discomfort were observed. Animal studies were conducted by the Guidelines of the institution’s Animal Care and Use Committee.

### Statistical analysis

GraphPad Prism was used for data analysis, and data were presented as means ± SEM from at least three biological replicates. When comparing vehicle control groups with treatment groups, an unpaired Student’s t-test was used in this study. *P* < 0.05 was considered to be statistically significant.

## Results

### Piceatannol promotes autophagy flux

**Figure 1:**
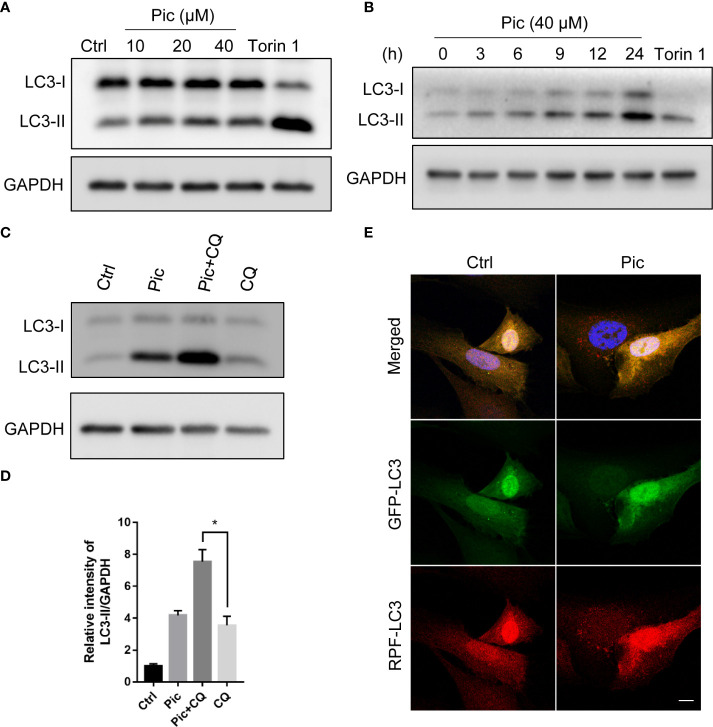
Piceatannol promotes autophagy flux. **(A)** Piceatannol (Pic) dose-dependently increases LC3-II levels. U2OS cells were treated with indicated concentration of Pic for 24 h, the expression of autophagy marker LC3-II was examined. **(B)** Pic time-dependently increases LC3-II levels. The expression of LC3-II was examined after treating cells with 40 µM Pic for indicated durations in U2OS cells. **(C, D)** Pic (40 µM) further increases LC3-II levels when in combination with lysosomal inhibitor chloroquine (CQ). **(E)** Pic increases autolysosomes. After transfected tf-LC3 plasmid into cells for 48 h, cells were incubated with Pic (40 µM) for another 24 h, the green and red-only puncta was examined by a fluorescence microscope **(E)**. Scale bar: 10 µm. Error bars indicate SEM for at least three biological replicates. Student-t test was used for statistical analysis. *p-value < 0.05.

To identify novel autophagy activators that can enhance the anticancer effects of chemotherapy-induced immunogenic cell death, we initially screened several natural dietary compounds that can induce autophagy in U2OS model cells as described previously (22). We discovered that treatment of cells with a dietary polyphenol piceatannol (Pic) for 24 h dose-dependently (0 µM, 10 µM, 20 µM, 40 µM) increased LC3-II levels, a canonical marker of autophagy (**Figure 1A**), without obvious cytotoxicity. In addition, Piceatannol also upregulated LC3-II levels in a time-dependent manner (0 h, 3 h, 6 h, 9 h, 24 h) (**Figure 1B**). Expectedly, an established mTORC1 inhibitor Torin 1, which was a positive control, also increased LC3-II levels (**Figures 1A, B**). Next, we examined whether piceatannol could enhance autophagy flux by adding lysosomal inhibitor chloroquine (CQ). As shown in **Figures 1C, D**, piceatannol further increased LC3-II levels when in combine with CQ. Immunostaining results further confirmed that piceatannol significantly increased autolysosomes in cells transiently expressing tf-LC3 (**Figure 1E**). Altogether, these findings indicate that piceatannol enhances autophagy flux.

### Piceatannol promotes autophagy *via* TFEB/TFE3

**Figure 2:**
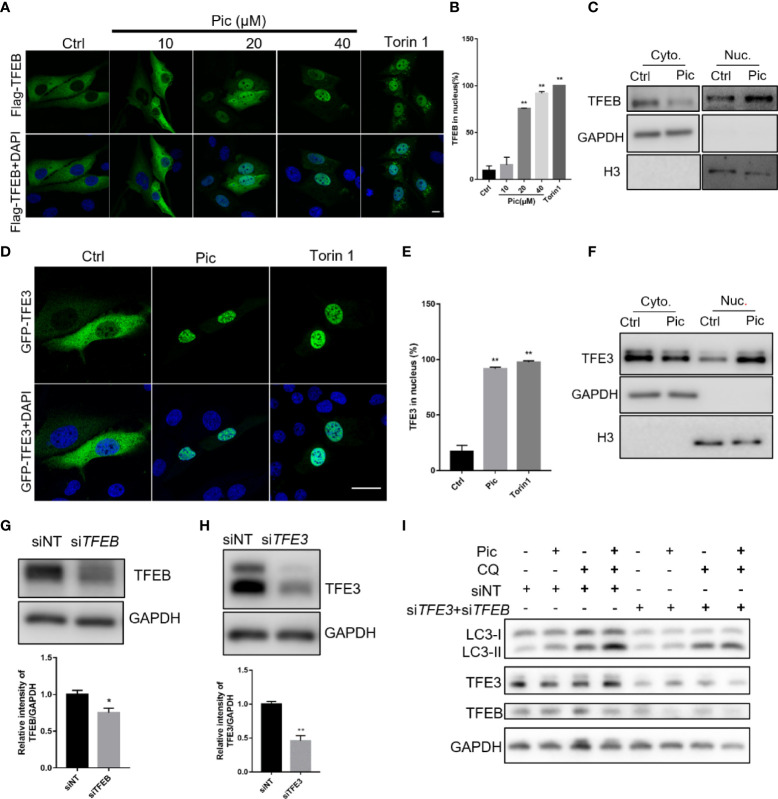
Piceatannol promotes autophagy flux *via* TFEB/TFE3. **(A, B)** Pic promotes the nuclear accumulation of TFEB. U2OS cells expressing Flag-TFEB were treated with indicated concentrations of Pic for 24 h before visualization of the nuclear accumulation of TFEB by immunostaining **(A)**, and the percentage of cells containing nuclear TFEB was quantified in **(B)**. Scale bar: 10 µm. **(C)** Pic-promoted nuclear accumulation of TFEB was further confirmed by western blotting in the cytoplasm and the nuclear fractions after treating cells with 40 µM Pic for 24 h. **(D, E)** Pic promotes the nuclear accumulation of TFE3. The nucleus accumulation of TFE3 was detected by immunostaining **(D)** and quantified **(E)** in U2OS cells expressing EGFP-TFE3 after incubating cells with 40 µM Pic for 24 h. Scale bar: 10 µm. **(F)** Western blotting analysis of the nuclear accumulation of TFE3 in the cytoplasm and the nuclear fractions from Pic (40 µM)-treated cells. **(G–I)** Knocked down the expression of TFEB **(G)** and TFE3 **(H)** inhibits Pic-induced autophagy flux **(I)**. Error bars indicate SEM for at least three biological replicates. Student-t test was used for statistical analysis. *p-value < 0.05 and **p-value < 0.01.

Transcription factor EB (TFEB) and transcription factor binding to IGHM enhancer 3 (TFE3) are key regulators of the autophagy-lysosome pathway. In the normal state, TFEB/TFE3 are retained in the cytoplasm in an inactivated state. Upon activation, TFEB/TFE3 translocate into the nucleus to upregulate multiple genes involved in autophagy. We next examined whether piceatannol induces autophagy *via* TFEB/TFE3. We transfected Flag-tagged TFEB (**Figure 2A**) and GFP-tagged TFE3 (**Figure 2D**) into U2OS cells before piceatannol treatment with indicated concentrations for 24 h. Immunostaining results showed that 20 µM and 40 µM piceatannol significantly increased the nuclear accumulation of TFEB (**Figures 2A, B**), and the percentage of cells containing nuclear TFEB in response to 40 µM piceatannol was similar to that upon Torin1 treatment, which was a positive control (**Figures 2A, B**). This result was further confirmed by detecting the expression of endogenous nuclear TFEB levels by western blotting analysis of the cytoplasm and the nuclear fractions (**Figure 2C**). We also found that piceatannol effectively promoted the nuclear accumulation of TFE3 as shown by immunostaining results (**Figures 2D, E**) and western blotting analysis (**Figure 2F**). We then knocked down the expression of TFEB and TFE3 by using specific siRNAs (**Figures 2G, H**), and we found that TFEB and TFE3 knockdown attenuated piceatannol-induced autophagy flux (**Figure 2I**). These findings indicate that piceatannol induces autophagy *via* TFEB/TFE3.

### Autophagy is required for the role of piceatannol in enhancing OXA-induced ATP release

**Figure 3:**
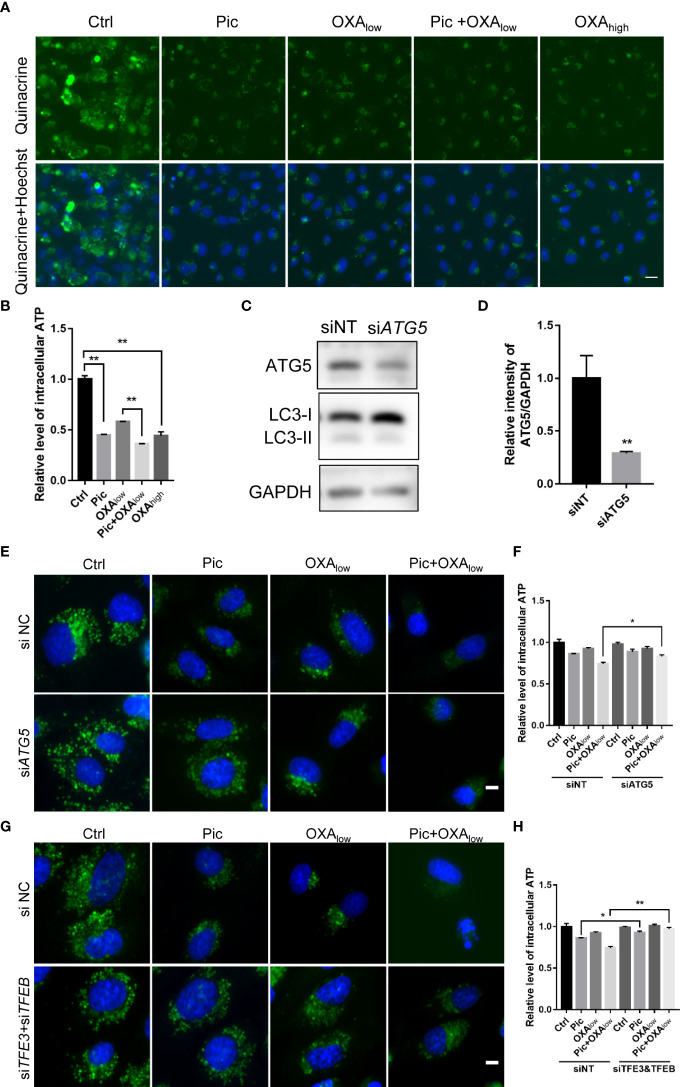
Piceatannol enhances ICD inducer OXA-induced ATP release *via* TFEB/TFE3-mediated autophagy. **(A, B)** Pic (40 μM) in combination with a low concentration of the ICD inducer oxaliplatin (OXA_low_, 200 μM) reduces intracellular ATP levels in U2OS cells. ATP was measured by quinacrine staining in cells treated with Ctrl, Pic, OXA_low_, OXA_low_+Pic and a higher concentration of OXA (OXA_high_, 400 μM), which was used as a positive control **(A)**. Scale bar: 20 μm. The intracellular ATP levels was quantified in **(B)**. **(C–F)** Pic (40 μM) plus OXA_low_-induced ATP release is comprised after knocking down the expression of an autophagy essential gene, ATG5. U2OS cells were transfected with ATG5 specific siRNA for 48 h before western blotting analysis of the levels of ATG5 and LC3-II **(C)**, the ratio of the relative intensity of ATG5 over that of GAPDH was quantified **(D)**. 48 h post si-ATG5 transfection, cells were treated with Pic (40 μm), OXA_low_ and OXA_low_+Pic for another 24h. The intracellular ATP levels were detected by quinacrine staining as in **(A, E)**, and the relative ATP level was quantified in **(F)**. Scale bar: 10 μm. **(G, H)** Pic (40 μM) plus OXA_low_-induced ATP release is compromised after knocking down the expression of TFEB and TFE3. The intracellular ATP levels were detected by quinacrine staining as in **(A, G)**, and the relative ATP level was quantified in **(H)**. Data were means ± SEM from at least three replicates. Student-t test was used for statistical analysis. *p-value < 0.05 and **p-value < 0.01.

Previous studies indicated that autophagy is required for the secretion of ATP, a key characteristic of ICD, from stressed/dying cells during ICD (10, 12). Because our results showed that piceatannol was an autophagy activator, we examined whether piceatannol could enhance the OXA-induced secretion of ATP. We used quinacrine, a fluorescent probe, to visualize released ATP levels. Our results showed that piceatannol reduced the intracellular abundance of quinacrine-positive ATP levels, and further reduced intracellular ATP levels when combined with a low concentration of OXA (Pic+OXA_low_) (**Figures 3A, B**). Moreover, knockdown of the expression of ATG5 (**Figures 3C, D**), an essential gene for autophagy, inhibited piceatannol plus OXA_low_-induced ATP release (**Figures 3E, F**). Consistently, deficiency in both TFEB and TFE3 also inhibited piceatannol plus OXA_low_-induced ATP release (**Figures 3G, H**). Together, these results suggest that piceatannol amplifies the release of a key ICD marker ATP, in the context of low-concentration of OXA in a TFEB/TFE3-autophagy-dependent manner.

### Piceatannol amplifies OXA-induced HMGB1 release and cell surface exposure of CALR

**Figure 4:**
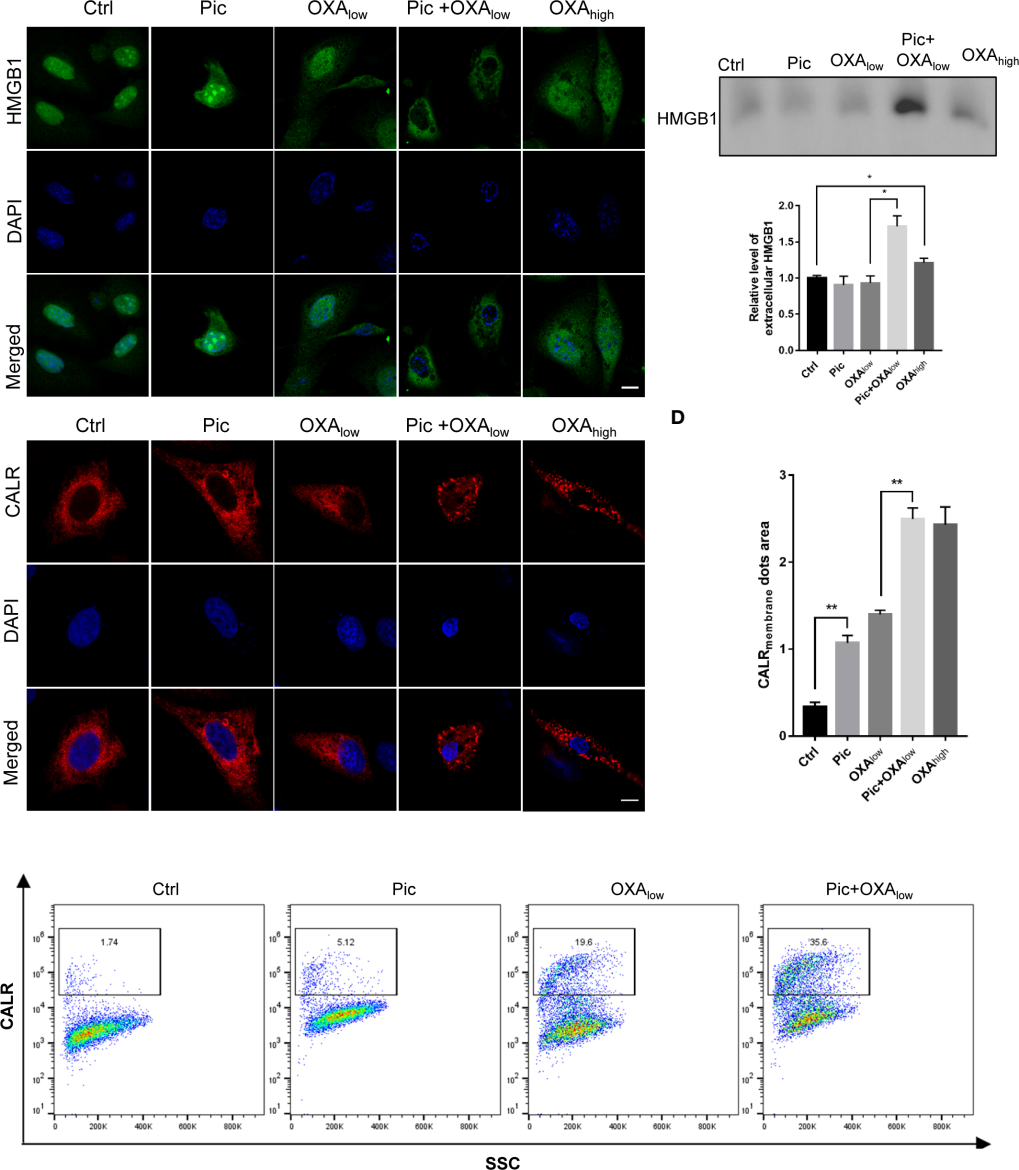
Piceatannol enhances ICD inducer OXA-induced high-mobility group box 1 (HMGB1) release and cell surface calreticulin (CALR) exposure. **(A)** Pic promotes OXA_low_-induced decrease of the nuclear HMGB1 contents. U2OS cells were treated with Pic (40 μM), OXA_low_ (200 μM) alone or their combination (Pic+OXA_low_) for 24 h, followed by detection of endogenous HMGB1 using immunostaining. OXA_high_ (400 μM) was used as a positive control. Scale bar: 10 μm. **(B)** Pic plus OXA_low_ increases extracellular HMGB1 contents as reflected by western blotting. The supernatant of the culture from the similar treatment as in **(A)** was collect for western blotting analysis of released HMGB1 and the relative level of HMGB1 for indicated treatment was quantified. **(C, D)**Pic plus OXA at the same concentration as in **(A)** increases the cell surface CALR exposure. U2OS cells transfected with CALR-RFP plasmid for 24 h were treated with Pic (40 μM), OXA_low_ (200 μM) alone or Pic+OXA_low_ for another 24 h before immunostaining detection **(C)** and the CALR membrane dots area was quantified in **(D)**. Scale bar: 10 μm. **(E)** Flow cytometry analysis further confirms the synergistic effect of Pic and OXA in inducing endogenous cell surface exposure. Cells treated with Pic (40 μM), OXA_low_ (200 μM) alone or Pic+OXA_low_ for 24 h were collected and stained with anti-CALR antibody. Data were mean ± SEM at least three replicates. Student-t test was used for statistical analysis. *p-value < 0.05, **p-value < 0.01.

We next determined the effects of piceatannol in enhancing OXA-induced other ICD hallmarks including HMGB1 release and cell surface exposure of CALR. We detected the changes of endogenous HMGB1 in response to piceatannol treatment for 24 h by Immunostaining. We found that piceatannol induced the release of endogenous HMGB1 from the nucleus to the plasma in combination with a low concentration of OXA in U2OS cells (**Figure 4A**). To detect the cytoplasmic HMGB1, we collected the supernatant from indicated cultures treated with piceatannol, OXA_low_, piceatannol+ OXA_low_ and OXA_high_, and precipitated the whole proteins for western blotting analysis. Consistently, we found that piceatannol plus low concentration of OXA significantly promoted the accumulation of extracellular release of HMGB1 (**Figure 4B**).

Since CALR can translocate from ER to the cell surface in response to several ICD-inducers, such as oxaliplatin and anthracyclines, we next examined whether piceatannol could promote OXA_low_-induced translocation of RFP-CALR from ER to the periphery of the cells. We transfected U2OS cells with Calreticulin-RFP-KDEL (21) plasmid and performed immunofluorescence staining, which showed that piceatannol in combination with OXA_low_ significantly enhanced the cell surface exposure of CALR to a similar level as that of high dose OXA (OXA_high_) treatment (**Figures 4C, D**). Flow cytometry analysis further confirmed the synergistic effect of piceatannol and OXA_low_ in improving the cell surface CALR exposure, compared to every single treatment with either piceatannol or OXA_low_ (**Figures 4E**). Altogether, our results suggest that piceatannol enhances ICD markers in combination with a low concentration of OXA.

### Piceatannol induces ER stress, which is involved in piceatannol plus OXA-induced cell surface CALR exposure

**Figure 5:**
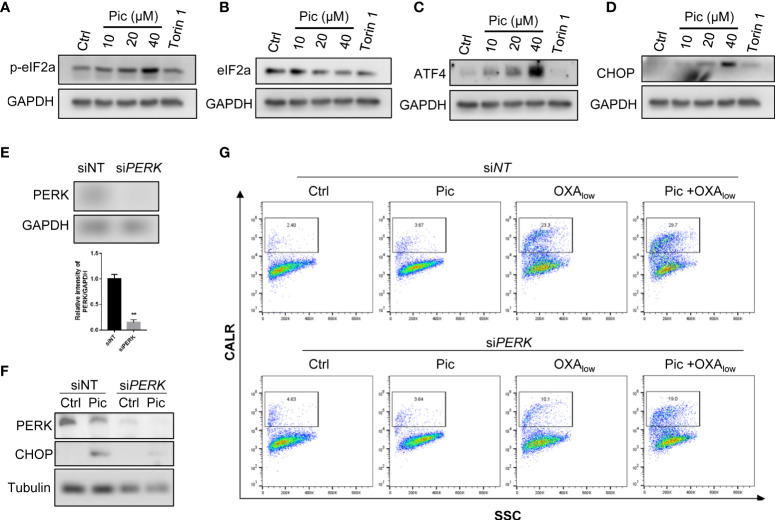
ER stress-mediated PERK activation is required for the role of Pic in enhancing OXA-induced CALR exposure. **(A, B)** Pic increases the phosphorylation of eIF2α. U2OS cells were treated with Pic (10-40 μM) for 24 h, p-eIF2α **(A)** and total eIF2α **(B)** were detected by western blotting. Torin 1 treatment was use as a positive control. **(C, D)** Pic increases the expression of canonical ER stress marker ATF4 and CHOP. U2OS cells were incubated with Pic (10-40 μM) for 24 h, ATF4 **(C)** and CHOP **(D)** were detected by western blotting. **(E, F)** knocked down the expression of PERK attenuates Pic-induced increasing of ER stress marker CHOP. U2OS cells were transfected with PERK-specific siRNA for 48 h **(E, F)**, and then treated with Pic (40 μM) for another 24 h, and the expression of CHOP was detected **(F)**. **(G)** Knockdown of the expression of PERK attenuates the combination effect of Pic and OXA in promoting cell surface CALR exposure. Cell surface CALR exposure was measured by Flow cytometry analysis. **p-value <0.01.

CALR is an ER luminal protein that is relocated to the cell peripheral during ICD, and ER stress-activated PERK-dependent eIF2α phosphorylation axis is involved in this process (23). Because our results showed that piceatannol increased OXA-induced cell surface exposure of CALR (**Figure 4**), we then determined whether piceatannol induces ER stress. As shown in **Figures 5A, B**, piceatannol increased the phosphorylation of eIF2α (p-eIF2α), a key event during ER stress. Phosphorylated eIF2α represses overall protein synthesis but upregulates the expression of ATF4, a key transcription factor of the integrated stress response. Consistently, we also found that piceatannol increased the levels of ATF4 (**Figure 5C**) and its downstream target CHOP (**Figure 5D**). These results suggest that piceatannol activates the eIF2α/ATF4/CHOP branch of unfolded protein response (UPR) (24). Previous studies have indicated that ER stress-induced eIF2α/ATF4/CHOP axis rather than other breaches of UPR contributes to CALR exposure in the context of ICD (25), thereby we further determined whether inhibition of p-eIF2α by knocking down the expression of PERK, a key kinase that phosphorylates eIF2α, attenuates piceatannol plus OXA-induced cell membrane exposure of CALR. Knocking down the expression of PERK (**Figures 5E, F**) indeed attenuated piceatannol-induced upregulation of CHOP (**Figure 5F**). Importantly, PERK deficiency compromised the synergistic effect of piceatannol and OXA-induced cell surface exposure of CALR (**Figure 5G**). These findings highlight the crucial role of ER stress-activated PERK/p-eIF2α axis in mediating the synergistic effect of piceatannol and OXA in enhancing cell surface exposure of CALR.

### Piceatannol amplifies OXA-induced ICD surrogate markers in MCA205 cells

**Figure 6:**
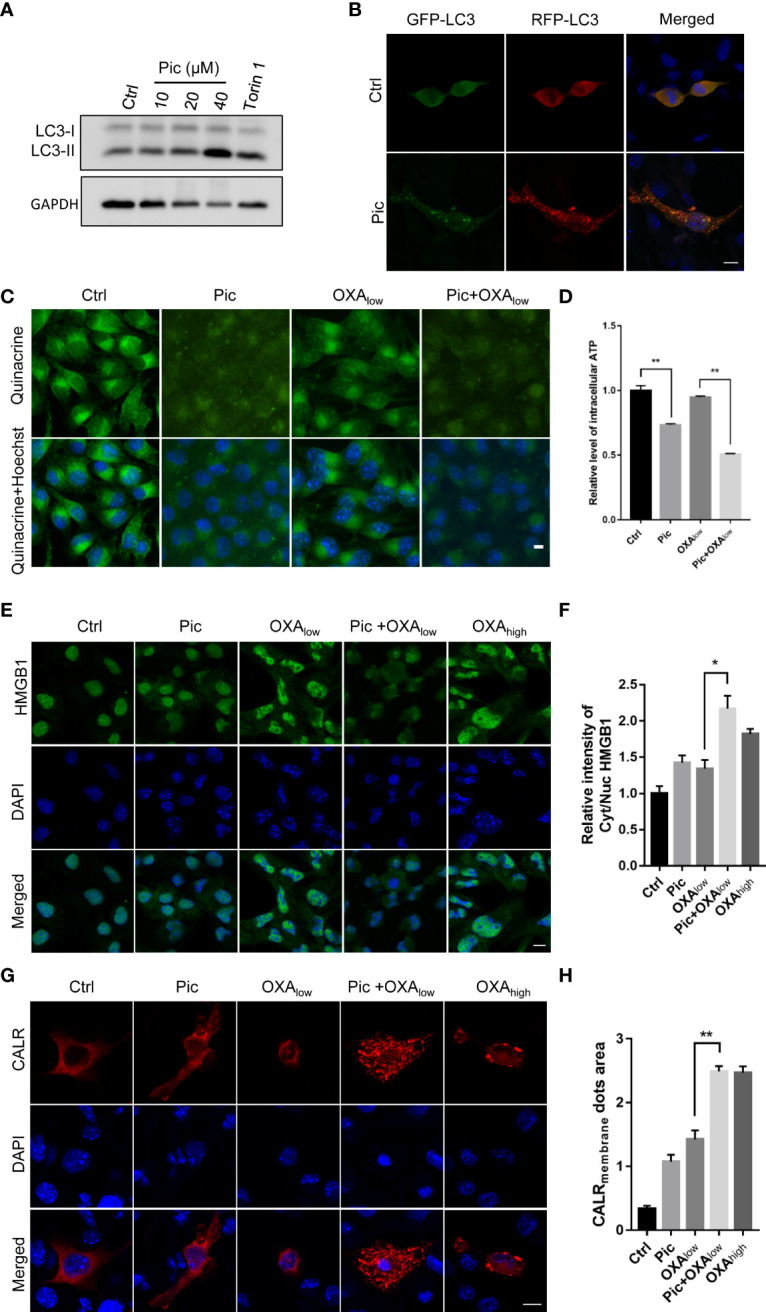
Pic increases autophagy and enhances OXA-induced ICD in MCA205 fibrosarcomas. **(A)** Pic increases LC3-II levels in MCA205 cells. MCA205 cells were treated with different concentrations of Pic (0, 10, 20 and 40 μM) for 24 h before western blotting analysis of LC3-II levels. **(B)** Pic increases autolysosomes as reflected by immunostaining in MCA205 cells expressing tf-LC3. Scale bar: 10 μm. **(C, D)** Pic (40 μM) reduces the intracellular ATP levels in MCA205 cells with OXA_low_ (200 μM) for 24 h measured by quinacrine staining. Scale bar: 10 μm. **(E, F)** Pic (40 μM) promotes the release of intracellular HMGB1 in combination with OXA_low_ for 24 h measured by immunostaining. Scale bar: 10 μm. **(G, H)** Pic (40 μM) promotes cell surface CALR exposure in combination with OXA_low_ for 24 h measured by immunostaining. Scale bar: 10 μm. Data were mean ± SEM of three replicates. Student-t test was used for statistical analysis. *p-value < 0.05, **p-value < 0.01.

To further evaluate if piceatannol can induce ICD hallmarks in other cancer cell types, we further determined its effects in MCA205 fibrosarcoma cells. In accordance with our findings in U2OS cells, piceatannol increased LC3-II levels (**Figure 6A**) and increased lysosomes in the tf-LC3 assay (**Figure 6B**), suggesting that piceatannol also enhances autophagy flux in MCA205 cells. Moreover, piceatannol significantly reduced the intensity of intracellular quinacrine (indictive of ATP levels), which was lower than that did by OXA_low_. Expectedly, the combination treatment with piceatannol and OXA_low_ further reduced the cytoplasm ATP levels (**Figures 6C, D**), indicating that piceatannol and OXA show additive effects in promoting the secretion of ATP. Additionally, piceatannol combined with OXA_low_ also enhanced HMGB1 release, and this effect was even stronger than that did by OXA_high_ (**Figures 6E, F**). Finally, our results showed that piceatannol combined with OXA_low_ increased the cell membrane exposure of CALR (**Figures 6G, H**). Altogether, these results demonstrate that piceatannol consistently amplifies OXA-induced hallmarks of ICD in fibrosarcoma cells as in U2OS cells, suggesting the conserved function of piceatannol in boosting OXA-induced ICD hallmarks in multiple cancer cells.

### Piceatannol increases the efficacy of OXA-induced ICD on immunogenic chemotherapy

**Figure 7:**
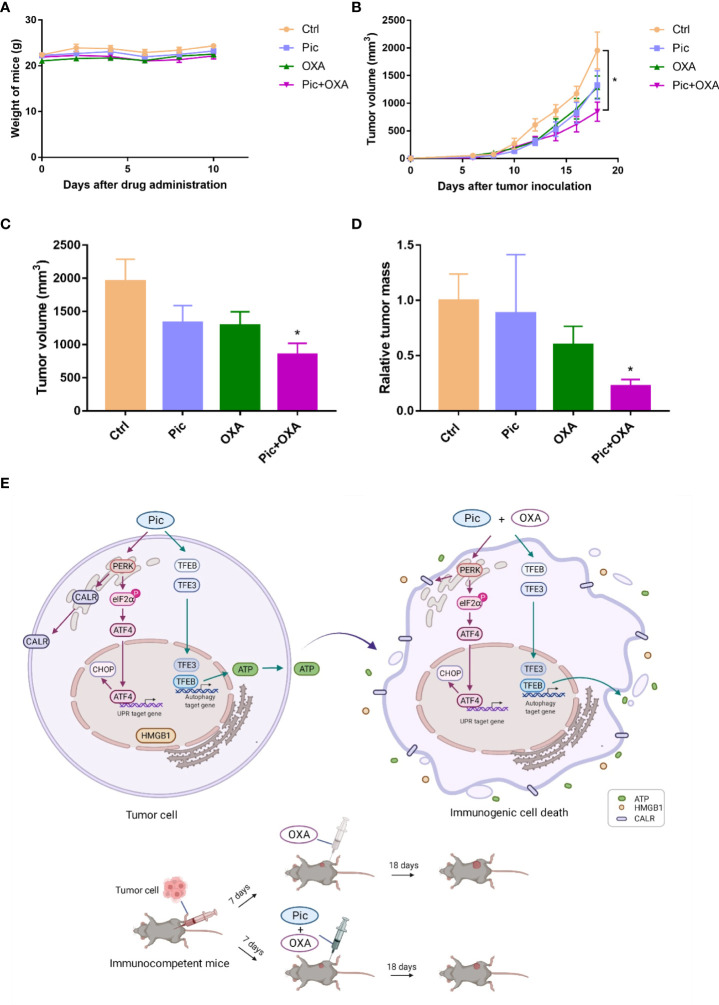
Piceatannol enhances the efficacy of anticancer chemotherapy of OXA in immunocompetent C57BL/6J mice. **(A)** Piceatannol (Pic), OXA_low_, or their combination (Pic+OXA_low_) does not obviously affect the body weight of C57BL/6J mice-bearing MCA205 tumor. **(B)** Growth curves (mean ± SEM) results show that Pic enhances the role of OXA_low_ in inhibiting tumor growth. **(C, D)** Pic enhances the role of OXA_low_ in reducing tumor volume **(C)** and weight **(D)**. **(E)** Schematic model showing the synergistic effect of Pic in enhancing low concentration of OXA-induced anti-tumor effect *via* amplifying ICD hallmarks. Mechanistically, Pic activated TFEB/TFE3 to induce autophagy, which was critical for its role in inducing ATP release and synergizing with OXA to boost ATP release. In addition, Pic induced ER stress, and activated PERK-involved UPR response. Specifically, Pic increased the phosphorylation of eIF2α, and increased ATF4 and CHOP level. This activated PERK/p-eIF2α/ATF4 axis triggered low level of cell surface CALR exposure and further promoted OXA-induced high level of cell surface CALR exposure. Additionally, Pic also synergized with OXA to enhance HMGB1 release. Consequently, Pic enhanced OXA-induced anticancer activity in immunocompetent mice bearing tumor cells possibly *via* its synergistic effect with OXA in amplifying ICD hallmarks. Unpaired T-test is used for statistical analysis. *p-value < 0.05.

Finally, we examined the effects of piceatannol in improving the efficacy of OXA-induced chemotherapy in immunocompetent mice bearing MCA205 fibrosarcomas. This model is a well-established immunosurveillance model (26, 27), and MCA205 fibrosarcomas growth beneath the skin can also be regarded as orthotopic. After tumors were established in C57BL/6J mice, we then treated mice with OXA_low_, piceatannol (Pic, 30 mg/kg), and piceatannol plus OXA_low_ (Pic+OXA_low_), and negative control (Ctrl) group (received vehicle only). As shown in **Figure 7A**, piceatannol, OXA_low_ alone or their combination did not affect mice’s body weight, indicating that this treatment shows no obvious toxicity. Interestingly, OXA_low_ combined with piceatannol (Pic+OXA_low_) significantly inhibited tumor growth, while OXA_low_ or piceatannol alone had no or little effects in inhibiting tumor growth (**Figures 7B-D**). These results indicate that piceatannol enhances the anti-cancer effect of OXA *in vivo*.

## Discussion and conclusion

Here, we discovered that piceatannol activates TFEB/TFE3 to induce autophagy, which is critical for synergizing with OXA to induce ATP release. We showed that piceatannol induces ER stress, which contributes to improving OXA-induced cell surface exposure of CALR. Piceatannol also improved OXA-induced HMGB1 release. Overall, piceatannol enhanced OXA-induced ICD hallmarks and subsequently increased the anticancer activity of OXA in immunocompetent mice.

Piceatannol is one of the natural polyphenolic compounds present in multiple dietary foods such as grapes blueberries, and mushrooms (15, 17), and it is a natural hydroxylated analogue of resveratrol (28). Piceatannol has been widely investigated for its roles in preventing disease and promoting health through the enormous biological activities, such as antioxidant, anti-cancer, neuroprotective effects, and cardiovascular protective effects (29–31). However, the specific mechanisms underlie such biological activities of piceatannol are still not fully understood. Autophagy has been implicated in multiple diseases including cancer (32, 33), and neurodegeneration (11, 34–36). However, whether piceatannol could induce TFEB/TFE3 dependent autophagy and how piceatannol exerts its anti- and protective effects in the aforementioned diseases has not been elucidated yet. Moreover, molecular mechanisms of how piceatannol activates TFEB, whether dependent on mTORC1 inhibition or calcium-dependent calcineurin activation are also unclear.

With respect to the anticancer effects of piceatannol, previous studies have shown that it suppresses multiple cancers growth or has cytotoxic effects such as in bladder cancer, brain cancer, leukemia, and breast cancer (31). Whether piceatannol modulates the anti-tumor immunity effects in combination with chemotherapeutics such as OXA has not been exploited. Here, our results revealed that piceatannol had little or no effects in inhibiting the growth of osteosarcoma and fibrosarcoma cells but effectively induced TFEB/TFE3 dependent autophagy, which is critical for the release of low level of ATP and high level of ATP when it was combined with OXA. In addition, piceatannol induced ER stress through activating the PERK/eIF2α/ATF4 signaling pathway, which contributed to its role in improving OXA-induced cell surface exposure of CALR. Piceatannol also improved OXA-induced HMGB1 release. The synergistic effect of piceatannol and OXA in inducing ICD ultimately amplified the anti-cancer killing activity of OXA in immunocompetent mice tumor models (**Figure 7E**). Interestingly, piceatannol solely could not induce HMGB1 release, indicating that piceatannol has a selective role in regulating ICD hallmarks. These findings are consistent with previous results that other autophagy enhancers such as hydroxycitrate (13), thiostreptone (22), and 3,4-Dimethoxychalcone (37) enhanced the anticancer effects of ICD inducers without intrinsic anticancer properties. Since autophagy plays critical roles in multiple diseases including cancer (32, 33), and neurodegeneration (11, 34, 35), our finding that piceatannol activates autophagy in a TFEB/TFE3 dependent manner, may partially explain the multiple beneficial biological activities of piceatannol. However, additional investigations are required to verify these effects.

Though we found that piceatannol effectively enhanced TFEB/TFE3-mediated autophagy, future studies to dissect the role of the upstream signaling pathways such as calcium/calcineurin (18, 38), or mTORC1 signaling (19, 39) in mediating the synergistic effect of piceatannol with OXA is fundamentally required. It is possible that piceatannol activates TFEB/TFE3 *via* mTORC1 inhibition since previous studies have shown that piceatannol inhibits PI3k/Akt/mTOR signaling (40). We also showed that piceatannol activated ER stress, a critical step to induce cell surface exposure of CALR, a key hallmark of ICD. Since TFEB/TFE3 are known to trigger ER stress (41), and ER stress is also capable of inducing autophagy (42, 43), further studies to dissect the crosstalk between piceatannol-induced ER stress and TFEB/TFE3 are helpful for understanding the precise mechanism of piceatannol in enhancing the anticancer effects of ICD. Furthermore, apart from OXA, to investigate whether piceatannol enhances the efficacy of other immunogenic chemotherapy including methotrexate (MTX) is also an interesting direction because autophagy and ER stress are also involved. Importantly, exploring the *in vivo* anti-cancer effect of piceatannol by complemented with immune checkpoint blockade is also an interesting topic.

Taken together, we discover that the dietary compound piceatannol is a novel potent TFEB/TFE3-mediated autophagy enhancer, and it exerts a previous uncharacterized immune-mediated anti-tumor response by synergizing with immunogenic chemotherapeutic OXA. Hence, our study suggests a significant translational potential of piceatannol in increasing immunogenic chemotherapies.

## Data availability statement

The original contributions presented in the study are included in the article/**Supplementary Material**. Further inquiries can be directed to the corresponding author/s.

## Ethics statement

The animal study was reviewed and approved by Ethical Committee Board of the Shenzhen People’s Hospital.

## Author contributions

SW, GW, WW, and ZX performed most of the experiments. JY, and MC also contributed to partial of the experimental data. QW, JW, CY and WZ contributed to conception, design, and supervision of the study, and provided funding support. All authors interpreted and analyzed the data. WZ wrote the first draft of the manuscript, SW, GW, WW, and ZX wrote the material and methods sections of the manuscript. All authors listed have read, revised, edited, and approved the submitted manuscript, and they have made a substantial, direct, and intellectual contribution to the work. All authors agreed to be accountable for all aspects of the work ensuring integrity and accuracy, and approved it for publication.

## Funding

This work was supported by the National Natural Science Foundation of China (82003721), National Key Research and Development Program of China (2020YFA0908000), Shenzhen Science and Technology Innovation Commission (JCYJ20210324114014039, JCYJ20210324115800001), China Postdoctoral Science Foundation (2020M683182), and Guangdong Basic and Applied Basic Research Foundation (2020A1515110549).

## Conflict of interest

The authors declare that the research was conducted in the absence of any commercial or financial relationships that could be construed as a potential conflict of interest.

## Publisher’s note

All claims expressed in this article are solely those of the authors and do not necessarily represent those of their affiliated organizations, or those of the publisher, the editors and the reviewers. Any product that may be evaluated in this article, or claim that may be made by its manufacturer, is not guaranteed or endorsed by the publisher.
