# Supplementary material for: Autophagy activation by dietary piceatannol enhances the efficacy of immunogenic chemotherapy
Source: Front Immunol. 2022 Aug 1;13:968686. doi: 10.3389/fimmu.2022.968686 (PMC9376326; doi:10.3389/fimmu.2022.968686)
Supplement: Supplementary file 1 [file DataSheet_1.docx]

**Supplementary Figure 1, Original blot images with fully visible membrane edges**

**Figure 1A**


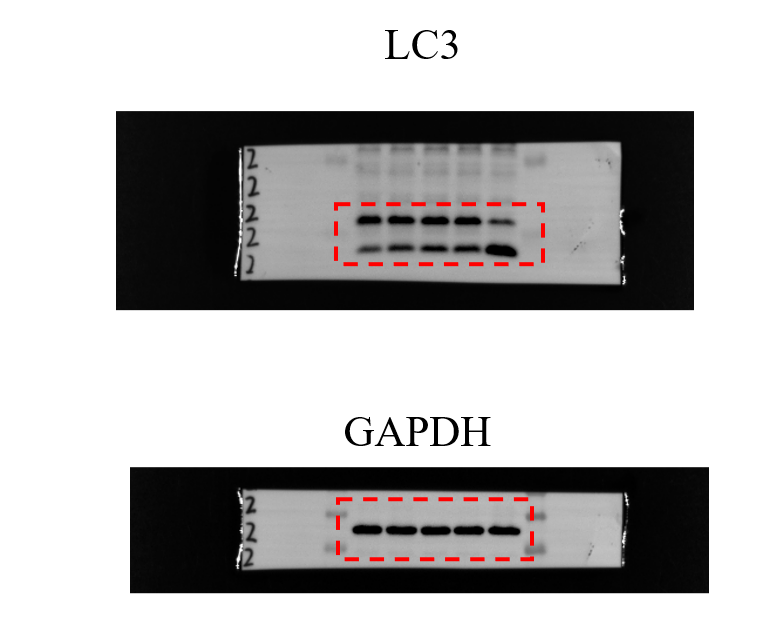


**Figure 1B**


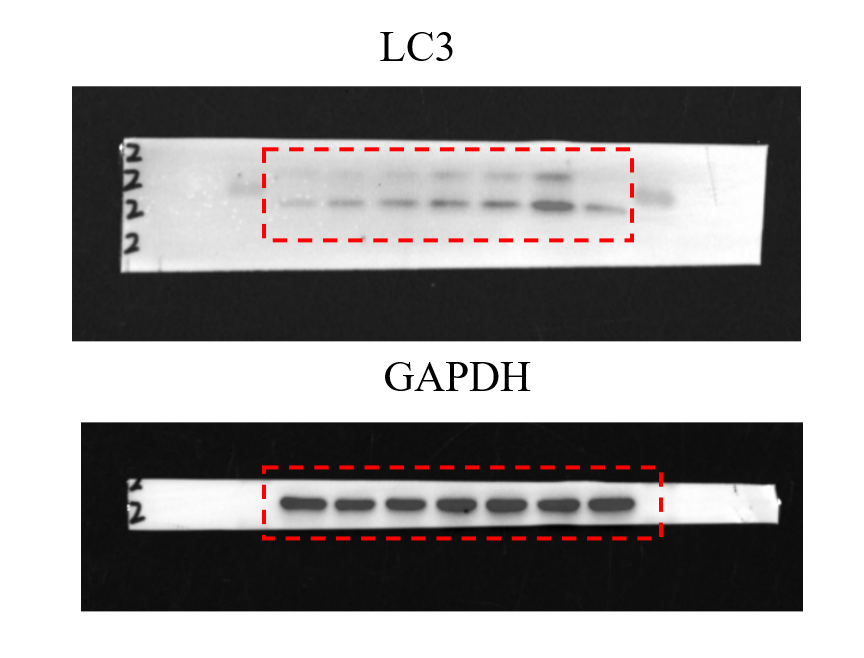


**Figure 1C**


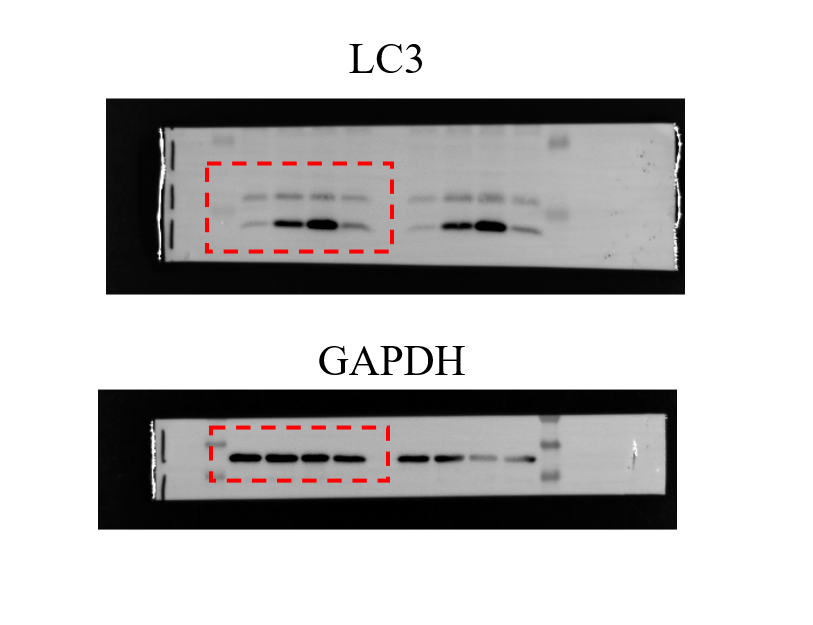


**Figure 2C**


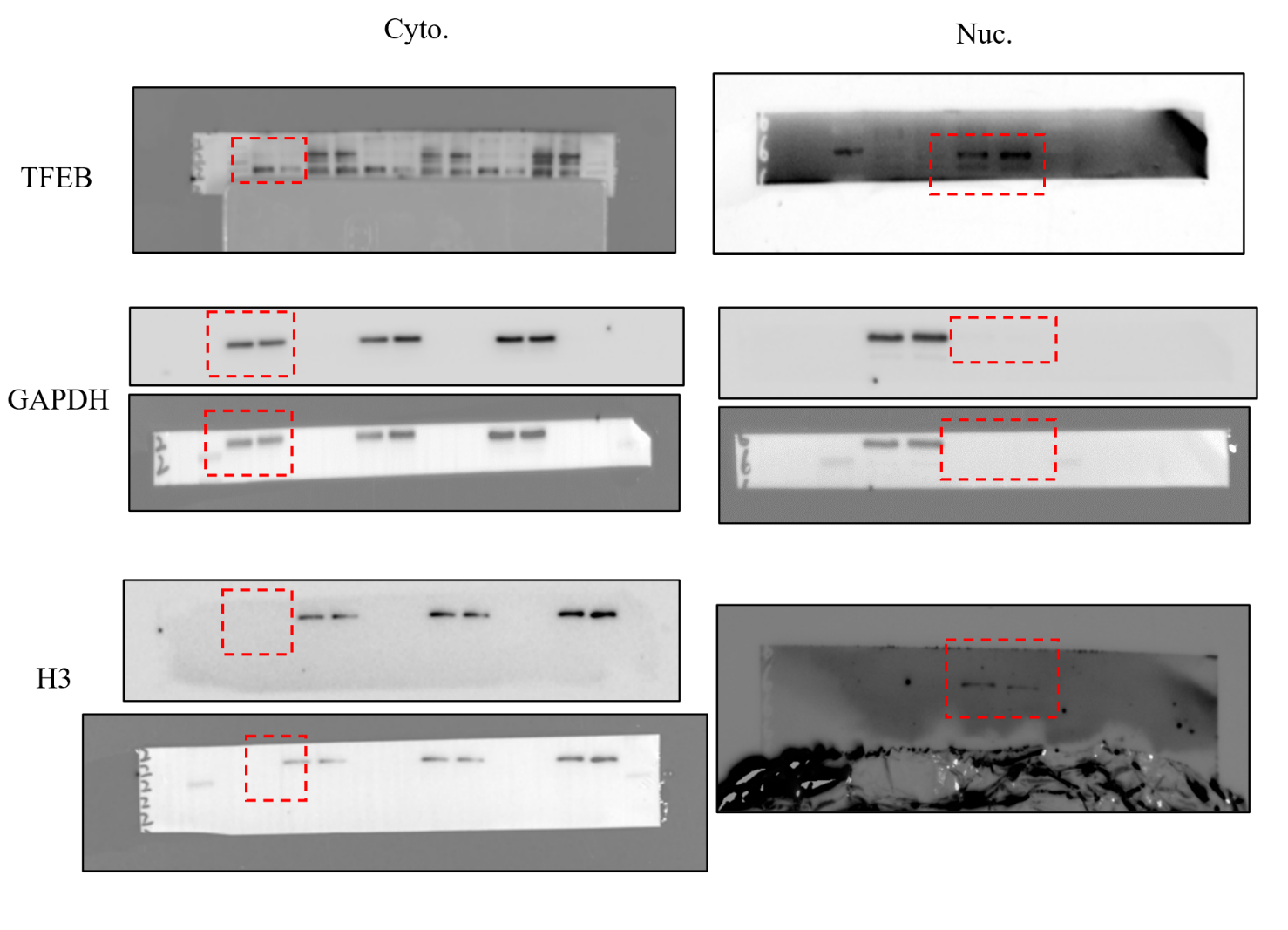


**Figure 2F**


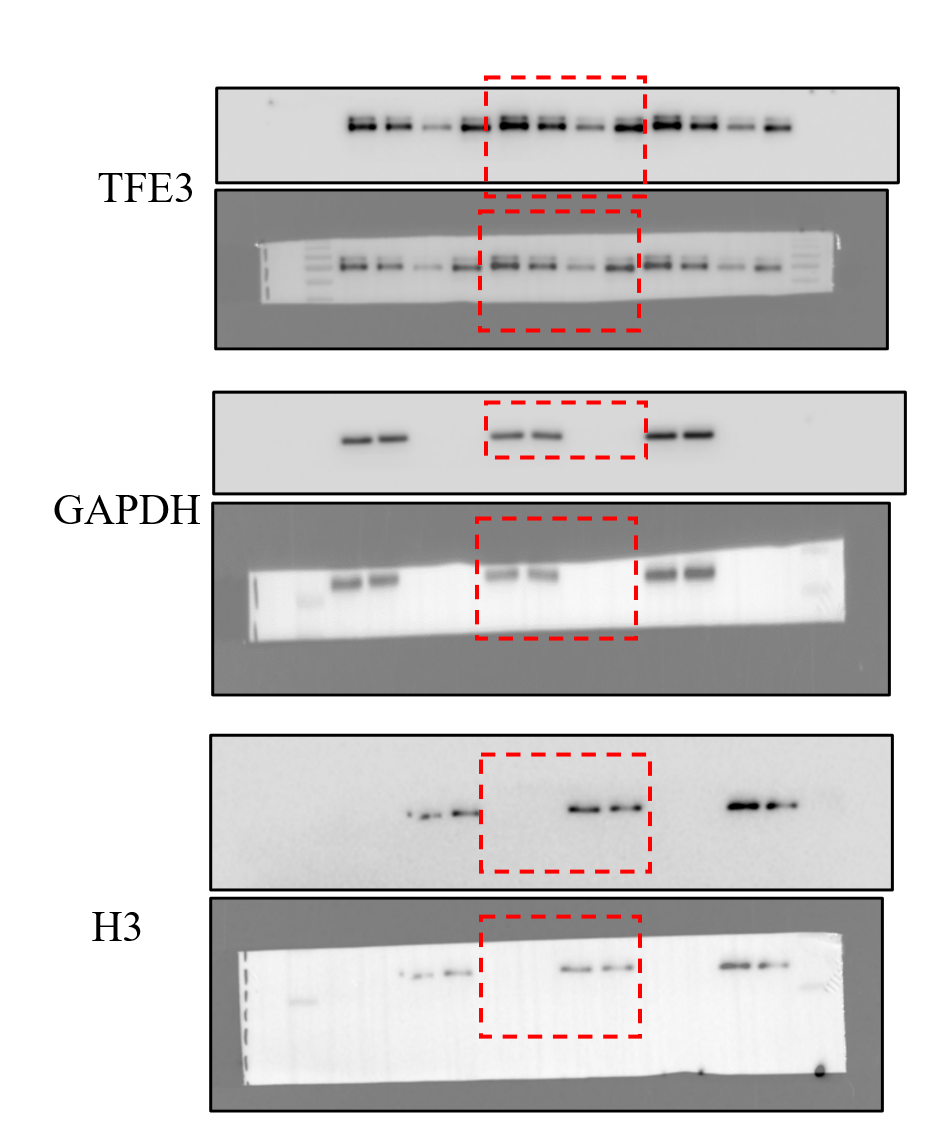


**Figure 2G**


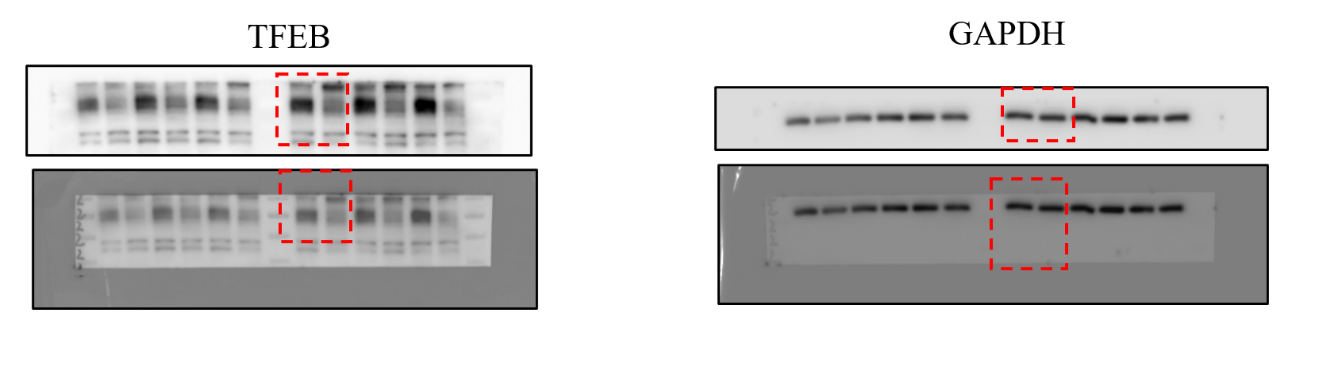


**Figure 2H**


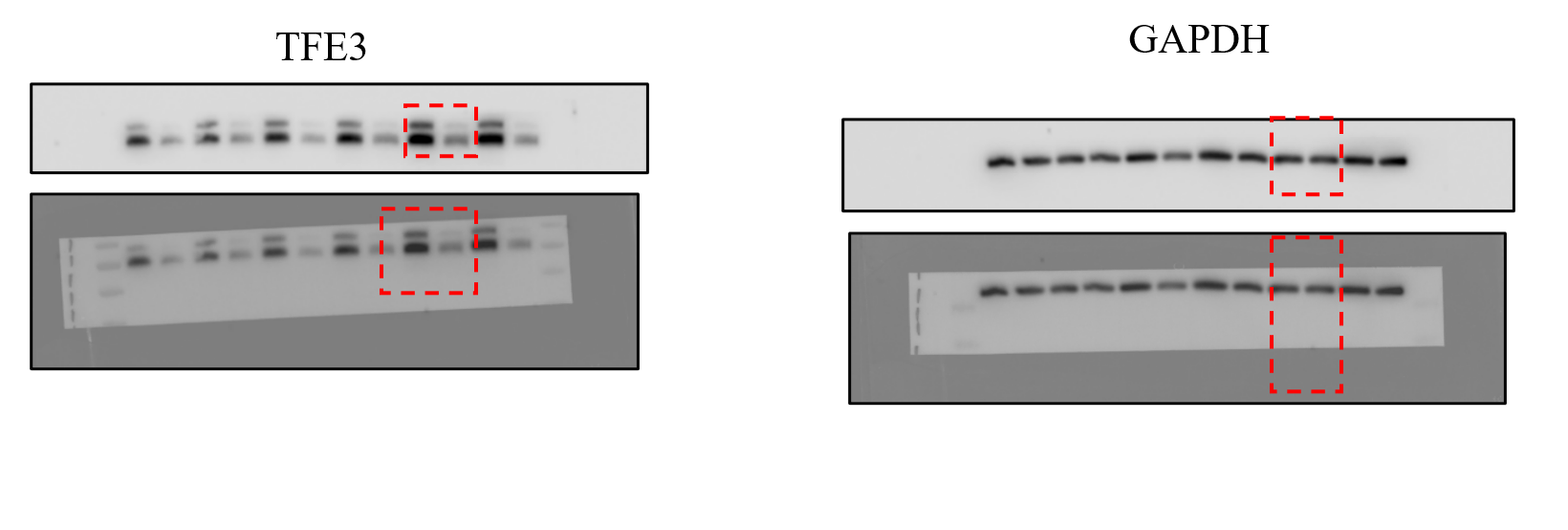


**Figure 2I**


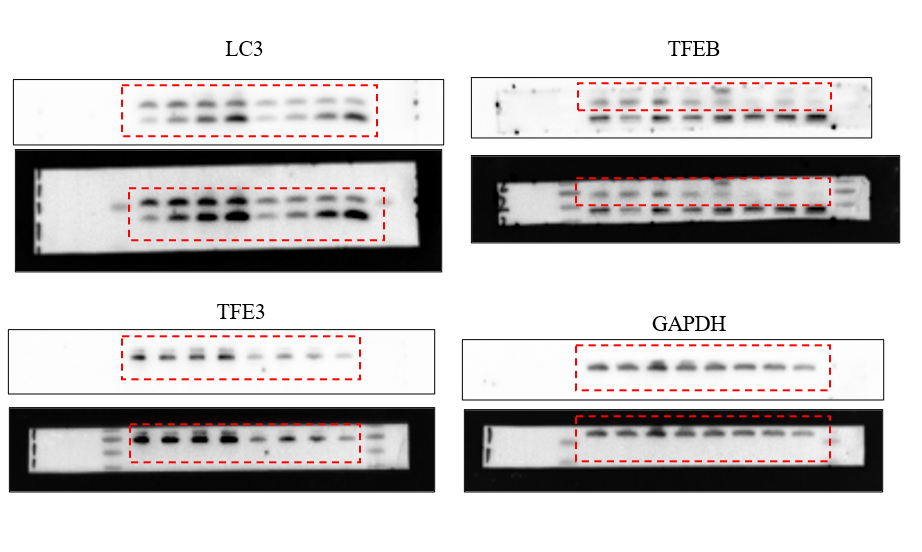


**Figure 3C**


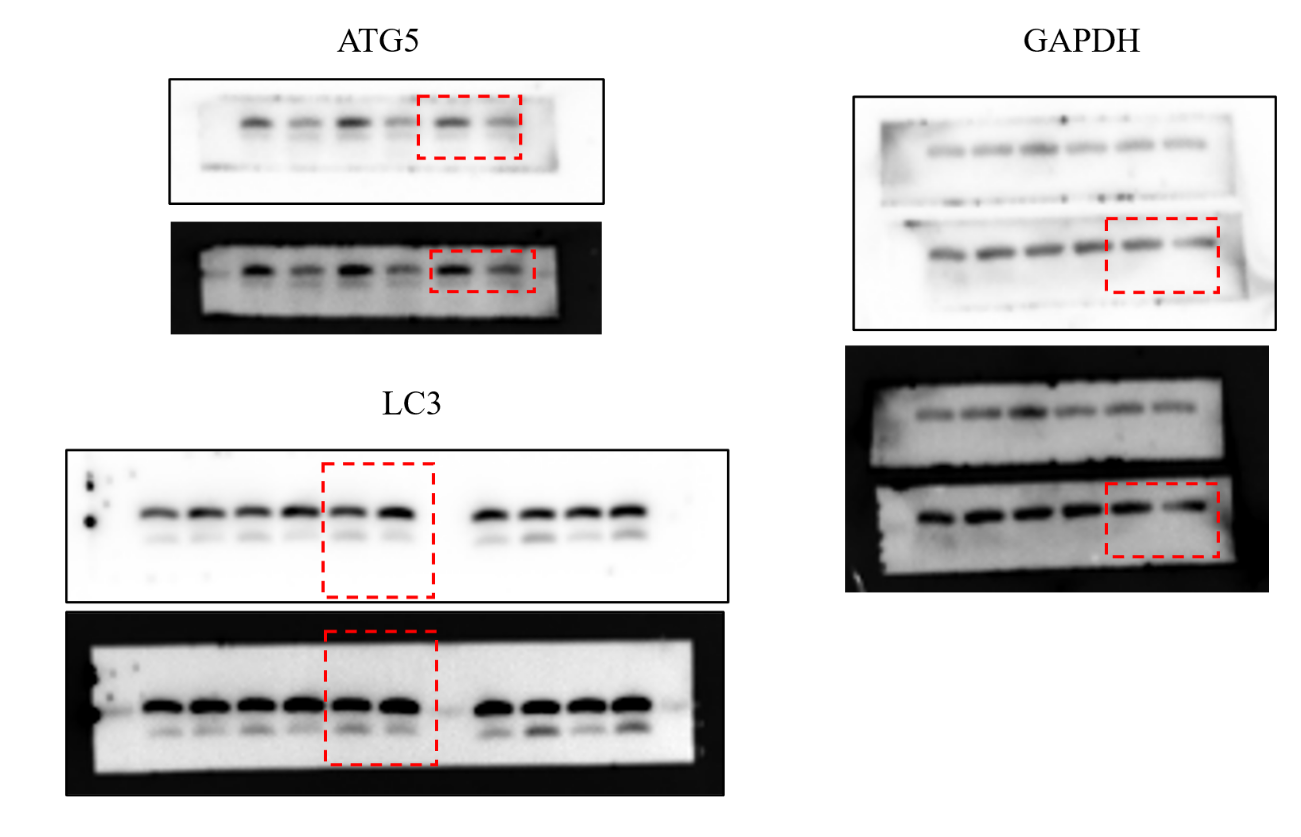


**Figure 4B**


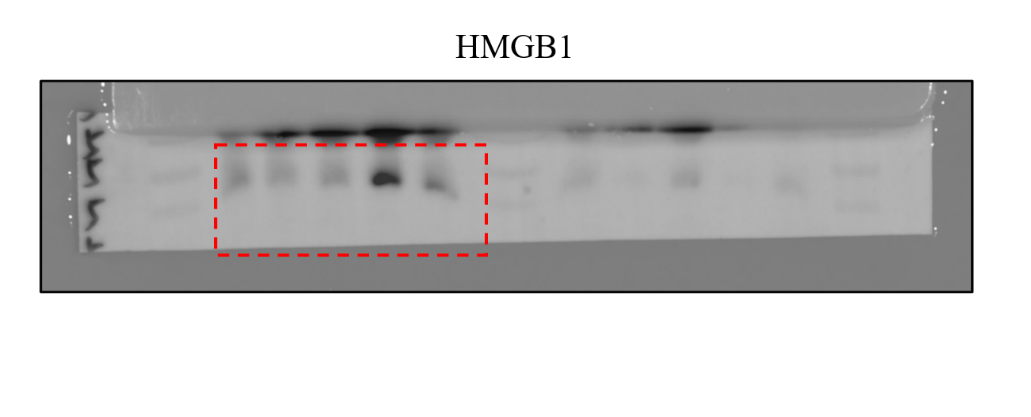


**Figure 5A**


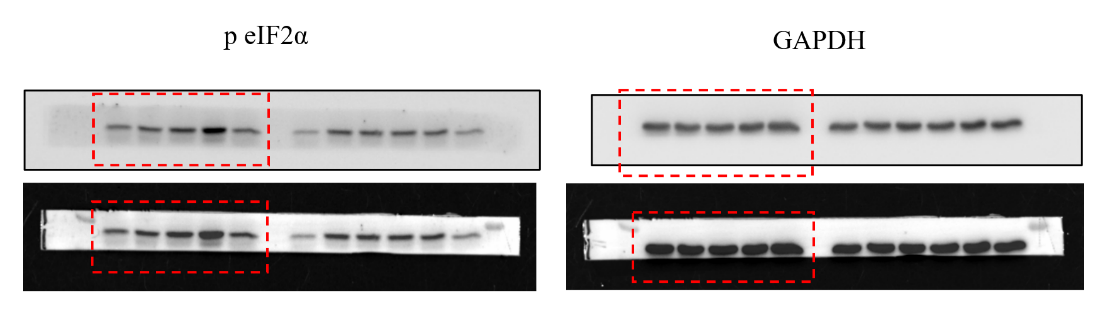


**Figure 5B**


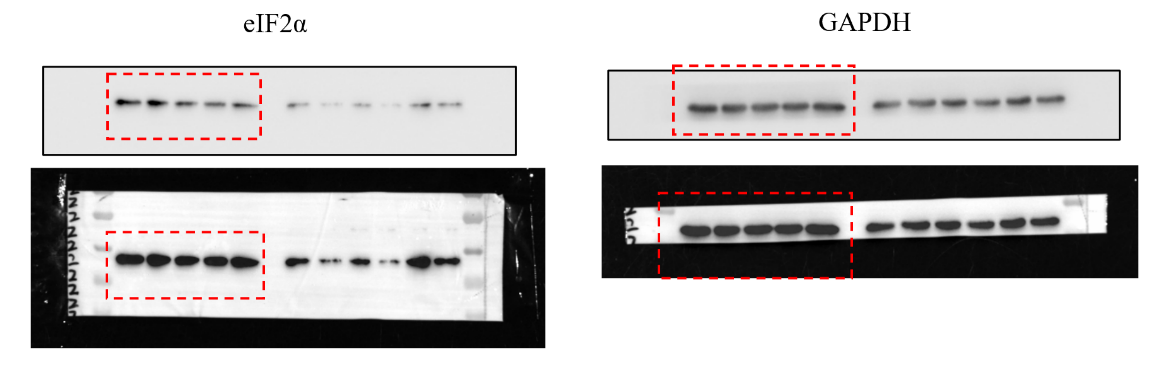


**Figure 5C**


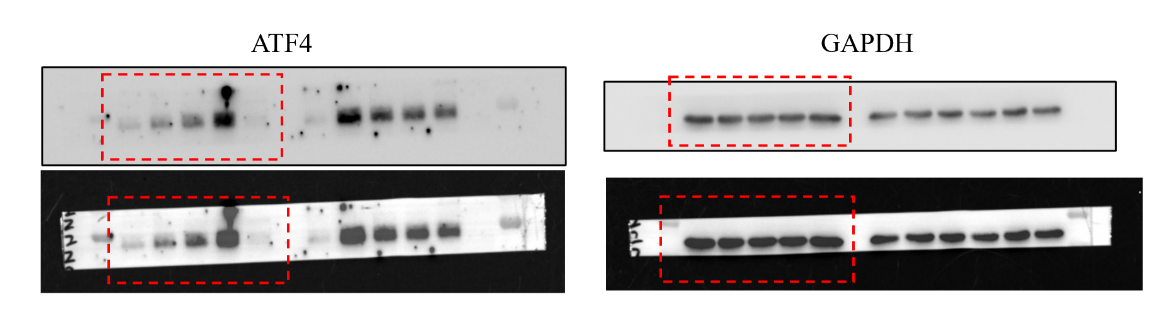


**Figure 5D**


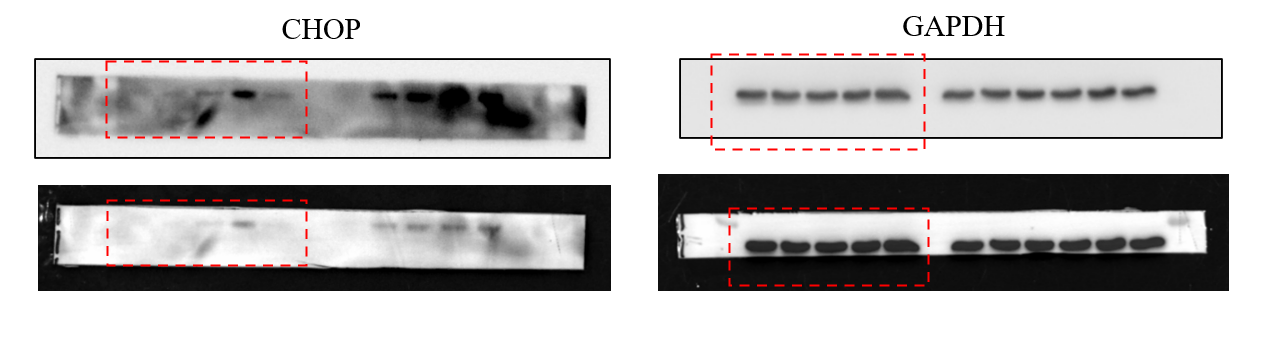


**Figure 5E**


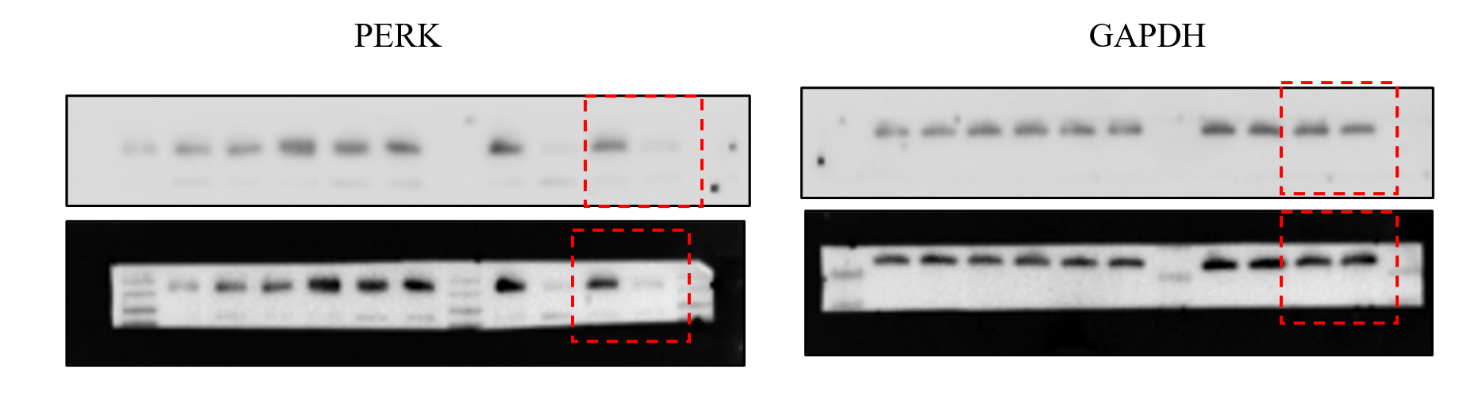


**Figure 5F**


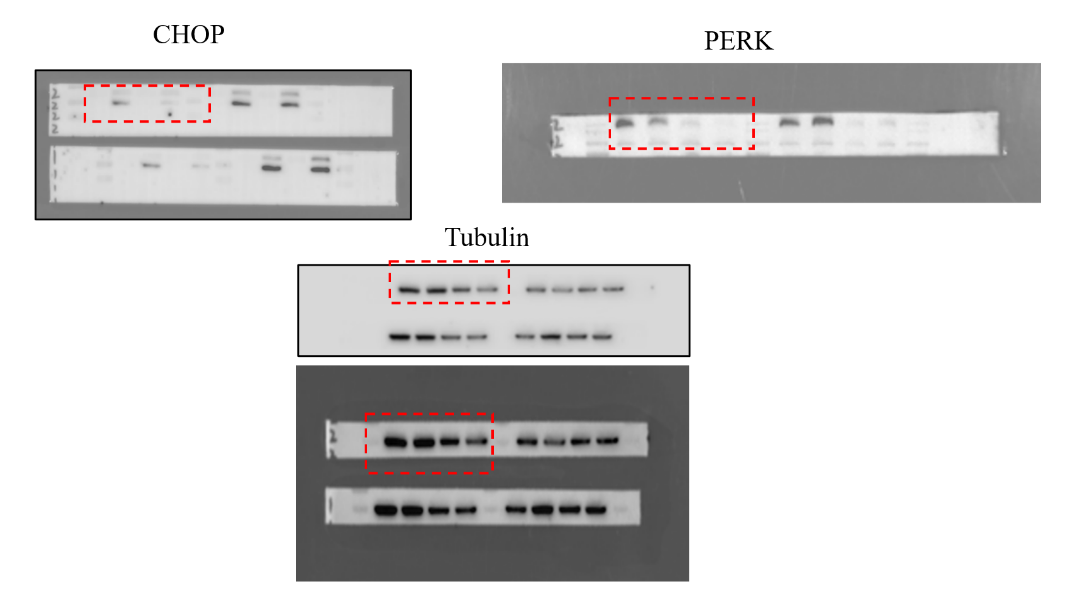


**Figure 6A**


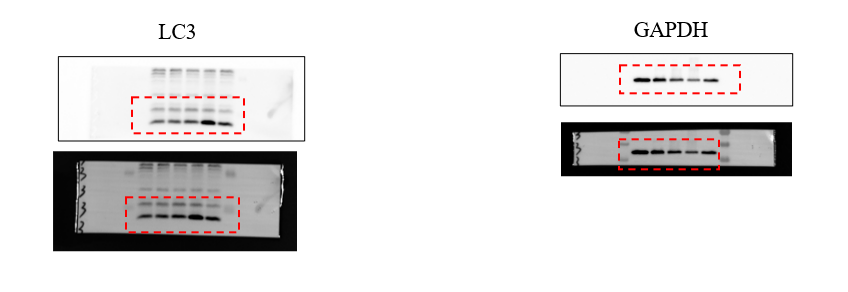
 **Supplementary Figure 1, Original blot images with fully visible membrane edges**
