# Supplementary material for: Autophagy activation by dietary piceatannol enhances the efficacy of immunogenic chemotherapy
Source: Front Immunol. 2022 Aug 1;13:968686. doi: 10.3389/fimmu.2022.968686 (PMC9376326; doi:10.3389/fimmu.2022.968686)
Supplement: Supplementary file 2 [file DataSheet_2.docx]

**Gating strategy for flow cytometry**


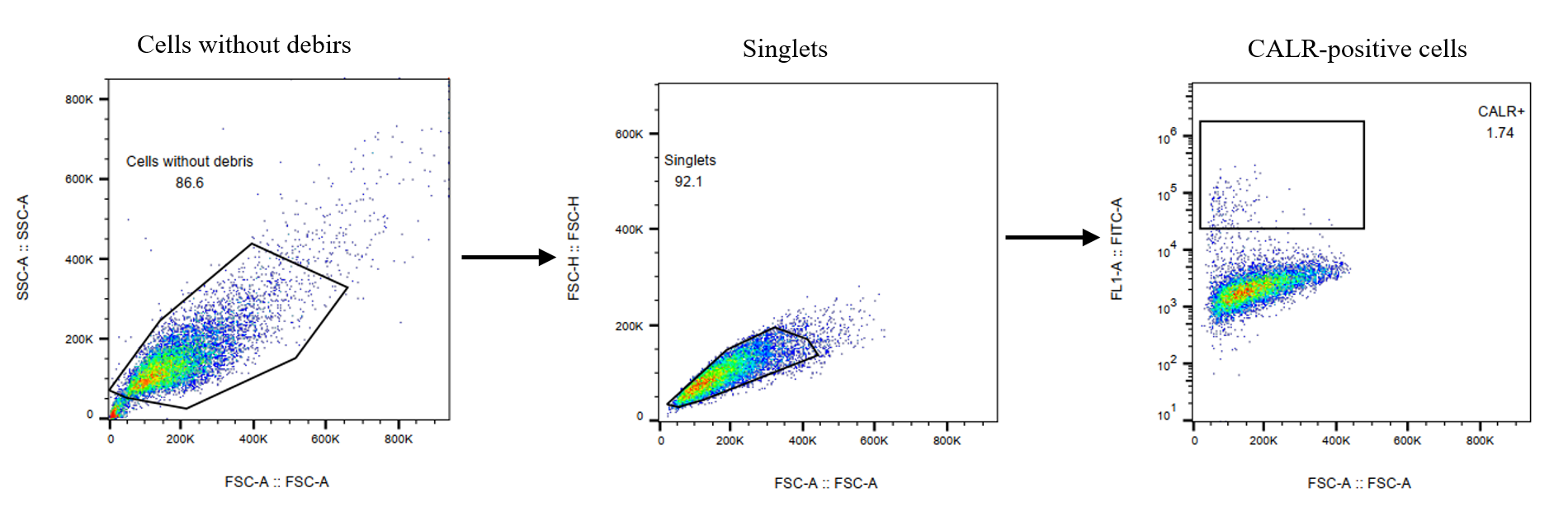


**Figure S2 Gating strategy for identification of CALR-positive cells in Fig. 4E and Fig. 5G.** Representative dot plots illustrate a gating strategy for the identification of CALR-positive cells.
